# Granulocyte-colony stimulating factor producing rectal cancer

**DOI:** 10.1186/1477-7819-6-70

**Published:** 2008-06-29

**Authors:** Hiroki Takahashi, Akira Yasuda, Nubuo Ochi, Masaki Sakamoto, Satoru Takayama, Takehiro Wakasugi, Hitoshi Funahashi, Hirozumi Sawai, Mikinori Satoh, Yoshimi Akamo, Hiromitsu Takeyama

**Affiliations:** 1 Department of Gastroenterological Surgery, Nagoya City University Graduate School of Medical Sciences, Kawasumi 1, Mizuho-cho, Mizuho-ku, Nagoya, Japan

## Abstract

**Background:**

Granulocyte-colony stimulating factor (G-CSF)-producing cancer has been reported to occur in various organs, especially the lung. However, G-CSF-producing colorectal cancer (CRC) has never been reported in the English literature.

**Case presentation:**

A 57-year-old man was admitted for the surgical removal of a rectal cancer. Some hepatic tumors in the liver were revealed concurrently, and their appearance suggested multiple liver metastases. Low anterior resection was performed. with the help of histopathological examination and immunohistochemical studies, we diagnosed this case to be an undifferentiated carcinoma of the rectum. After the operation, the white blood cell (WBC) count increased gradually to 81,000 cells/μL. Modified-FOLFOX6 therapy was initiated to treat the liver metastases, but there was no effect, and peritoneal dissemination had also occurred. The serum level of G-CSF was elevated to 840 pg/mL (normal range, <18.1 pg/mL). Furthermore, immunohistochemistry with a specific monoclonal antibody against G-CSF was positive; therefore, we diagnosed this tumor as a G-CSF-producing cancer. The patient died from rapid growth of the liver metastases and peritoneal dissemination 2 months after surgery.

**Conclusion:**

This is the first case of G-CSF-producing rectal cancer, and its prognosis was very poor.

## Background

Granulocyte-colony stimulating factor (G-CSF)-producing cancer has been reported to occur in the lung [1], stomach [2], esophagus [3], gall bladder [4], thyroid [5], urinary bladder [6], liver [7,8]. However, to the best of our knowledge, G-CSF-producing colorectal cancer (CRC) has never been reported in the English literature. G-CSF-producing cancers are thought to have a very poor prognosis. Furthermore, undifferentiated CRC is very rare and this is the first report of a G-CSF-producing undifferentiated cancer of the rectum. Its prognosis was very poor; therefore, we would like to report this case and discuss its clinicopathological features.

## Case presentation

**Figure 1:**
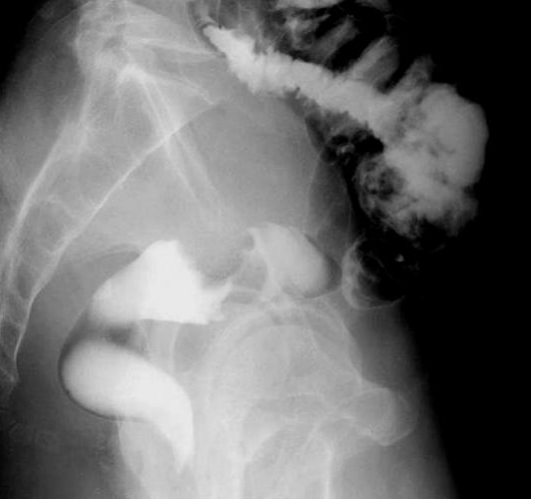
Barium enema revealed an ulcerative tumor in the rectum.

A 57-year-old man was admitted to our hospital with lower abdominal pain in June 2007. Barium enema and colonoscopy revealed an ulcerative tumor in the rectum (Figure 1), which, after biopsy, was diagnosed as a well differentiated adenocarcinoma. Physical examination showed no remarkable abnormalities. Neither hepatomegaly nor splenomegaly was apparent. Serum was negative for hepatitis B surface antigen and hepatitis C antibodies, and the patient had no history of alcohol intake or blood transfusion. Laboratory data on admission, including liver function tests, were unremarkable. The white blood cell (WBC) count was 8,000 cells/μL (neutrophil: 80.7%). Levels of carcinoembryonic antigen (CEA), carbohydrate antigen 19-9 (CA19-9), and α-fetoprotein (AFP) were within normal ranges. Abdominal ultrasonography showed a multiple hypoechoic, 1.5-cm diameter mass in the liver. Computed tomography (CT) and magnetic resonance imaging (MRI) with superparamagnetic iron oxide (SPIO) were performed, and the results suggested multiple liver metastases. The patient underwent low anterior resection on 25 July 2007.

**Figure 2:**
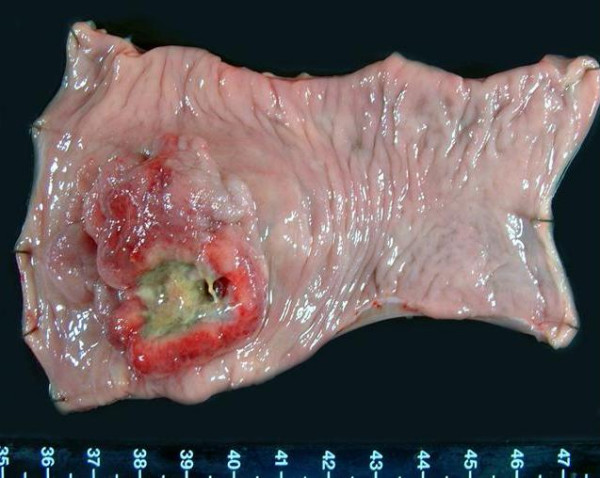
Resected specimen. An ulcerated hard tumor was present in the rectum.

**Figure 3:**
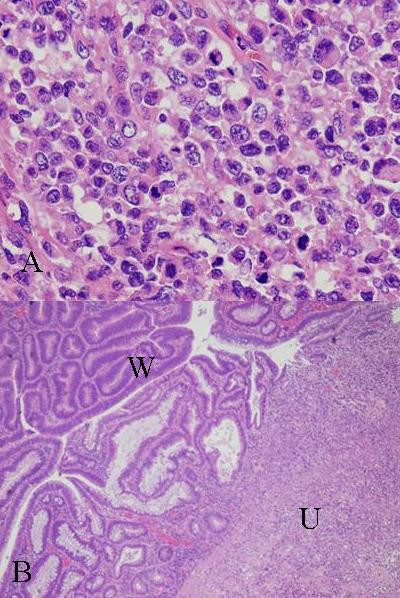
**HE staining**. a) Large abnormal cells without gland formation and mucin production were seen in the tumor (×400). b) A small component of well-differentiated adenocarcinoma was also seen on the surface of the tumor (W) amidst undifferentiated carcinoma (U) (×40).

**Figure 4:**
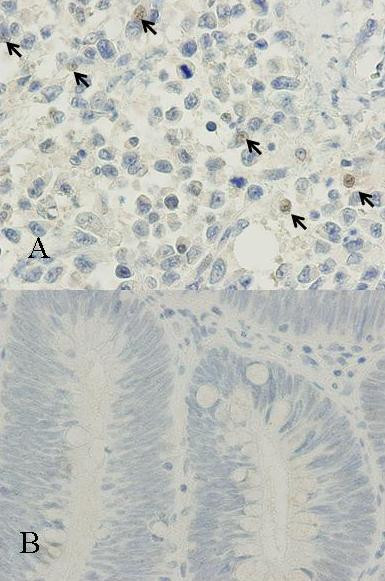
Immunohistochemical staining. a) Immunohistochemical staining with a specific monoclonal antibody against recombinant human G-CSF was positive in the cytoplasm of undifferentiated carcinoma cells (×400). b) The well-differentiated part of the adenocarcinoma was not stained with the G-CSF antibody (×400).

An ulcerated hard tumor was present in the rectum (Figure 2). Histopathological examination revealed that the tumor consisted of large abnormal cells without gland formation and mucin production (Figure 3a). Immunohistochemical studies were positive for cytokeratin and vimentin; however, they were negative for CD45 and PAS. In addition, they were negative for both neuron-specific enolase (NSE) and synaptophysin, and histologic staining with alcian blue was also negative. Therefore, we diagnosed this case to be an undifferentiated carcinoma of the rectum. A small component of well-differentiated adenocarcinoma was also seen on the surface of the tumor (Figure 3b). Thus, we thought that we diagnosed this tumor as well-differentiated adenocarcinoma at biopsy. Advanced lymphatic vessel and venous invasion were observed. Lymph node metastasis was also detected near the tumor, but peritoneal dissemination was not detected. After the operation, the WBC count gradually increased. Modified-FOLFOX6 (mFOLFOX6) therapy was initiated to treat the liver metastases, but it had no effect, and peritoneal dissemination occurred. Along with the growth of the tumor, the WBC count increased to 81,000 cells/μL (neutrophil: 87%). On the other hand, in comparison to the grade of leukocytosis, CRP level was not so high (6.5 mg/dl), and there were not any obvious signs of infection, so we suspected that this tumor produced G-CSF, and we measured serum G-CSF using an enzyme-linked immunosorbent assay (ELISA). The serum level of G-CSF was elevated to 840 pg/mL (normal range, <18.1 pg/mL). Furthermore, immunohistochemical staining with a specific monoclonal antibody against human G-CSF (11041, IBL, Gunma, Japan) was performed. G-CSF was positive in the cytoplasm of undifferentiated carcinoma cells (Figure 4a), but negative in the non-cancerous lesion and well-differentiated adenocarcinoma cells (Figure 4b). Unfortunately, we couldn't obtain biopsy specimens from the liver tumor. But, G-CSF was positive in metastatic lymph nodes, so we thought that G-CSF was also positive in the liver tumor. Therefore, we concluded that this tumor was a G-CSF-producing cancer. The patient died from rapid growth of the liver metastases and peritoneal dissemination 2 months after surgery.

## Discussion

G-CSF-producing cancer has been reported to occur in various organs, especially in the lung. Histologically, more than half of the reported cases of G-CSF-producing lung cancer have been large cell carcinoma [1]. However, it was reported that many cases of G-CSF-producing cancer of the digestive organs were poorly differentiated carcinoma or undifferentiated carcinoma [2,5,8,9]. Our case was mainly an undifferentiated carcinoma, so G-CSF-producing rectal cancer may have the same properties as other cancers of the digestive organs. However, an area of well-differentiated adenocarcinoma was seen on the surface of the tumor. Yamano *et al*., reported a case of early-stage gastric cancer that presented as a well-differentiated adenocarcinoma that changed to a poorly differentiated adenocarcinoma at the advanced stage, and acquired the ability to produce G-CSF [2]. In our case, G-CSF immunostaining was positive only in the undifferentiated cells and negative in the well-differentiated adenocarcinoma cells. Consequently, such histological changes might influence the ability to produce active G-CSF. Interestingly, it was reported that large cell carcinoma of the lung is often vimentin-positive [10], and our case was also vimentin-positive. Studies of other G-CSF-producing cancers have not investigated vimentin immunoreactivity, so further examination is required.

The mechanism by which certain CRCs produce G-CSF has not been clarified. Mroczko *et al*., reported that median values of G-CSF in CRC patients were significantly higher than those in healthy subjects [11]. Furthermore, it was reported that granulocyte-macrophage colony stimulating factor (GM-CSF) secretion was also detected by human colorectal cancer specimens and cell lines [12,13].

Tachibana *et al*., showed that G-CSF production by transitional cell carcinoma of the bladder augments autocrine growth, which may in part explain the poor prognoses [14]. Savarese **et al**., reported that 56.5% of primary ovarian carcinomas co-expressed G-CSF and the G-CSF receptor (G-CSFR); potential autocrine and/or paracrine loops involving G-CSF and its receptor occur in over 90% of primary ovarian carcinomas [15]. As mentioned earlier, some CRCs have the ability to secrete active G-CSF. Furthermore, Yang *et al*., reported that the G-CSFR was expressed in 59% of CRCs [16]. Therefore, autocrine growth is possible in CRC. Furthermore, Natori *et al*., reported that G-CSF stimulates angiogenesis and promotes tumor growth [17]. In addition, Tsuruta *et al*., showed that the production of GM-CSF by squamous cell carcinoma cell lines was closely related to their in vitro invasiveness and MMP activity [18]. For these reasons, G-CSF-producing cancer has a very poor prognosis, and at the present time, there is no specific approach for G-CSF-producing cancer. We performed mFOLFOX6 therapy to treat the liver metastases, but it had no effect, and the patient's general condition worsened rapidly. Usually, preoperative WBC count of G-CSF producing tumor is high and reduces after surgery or chemotherapy. In present case, preoperative WBC count was not so high and increased along with the growth of the tumor. We guess that this is because tumor volume was not large at the time of operation.

## Conclusion

In summary, we present the first case of G-CSF-producing rectal cancer. Its prognosis was very poor, and mFOLFOX6 therapy had no effect. But, we believe that further investigation may make it clear that molecular targeted therapies for G-CSF may become part of the treatment paradigm.

## Abbreviations

CRC: colorectal cancer; G-CSF: granulocyte-colony-stimulating factor; GM-CSF: granulocyte-macrophage colony stimulating factor; ELISA: enzyme-linked immunosorbent assay; G-CSFR: granulocyte-colony-stimulating factor receptor; CT: Computed tomography; MRI: Magnetic resonance imaging; mFOLFOX: Modified-FOLFOX6.

## Competing interests

The authors declare that they have no competing interests.

## Authors' contributions

HT, SM, NO, ST and HT carried out the clinical examination and operation, HT, AY, HS, and YA performed the pathological analysis, HT, HF and TW participated in the design of the study, HT and MS conceived the study, and participated in its design and coordination. All authors read and approved the final manuscript.
